# Supplementary material for: Identification and Validation of Novel Reference Genes in Acute Lymphoblastic Leukemia for Droplet Digital PCR
Source: Genes (Basel). 2019 May 17;10(5):376. doi: 10.3390/genes10050376 (PMC6562415; doi:10.3390/genes10050376)
Supplement: Supplementary file 1 [file genes-10-00376-s001.pdf]

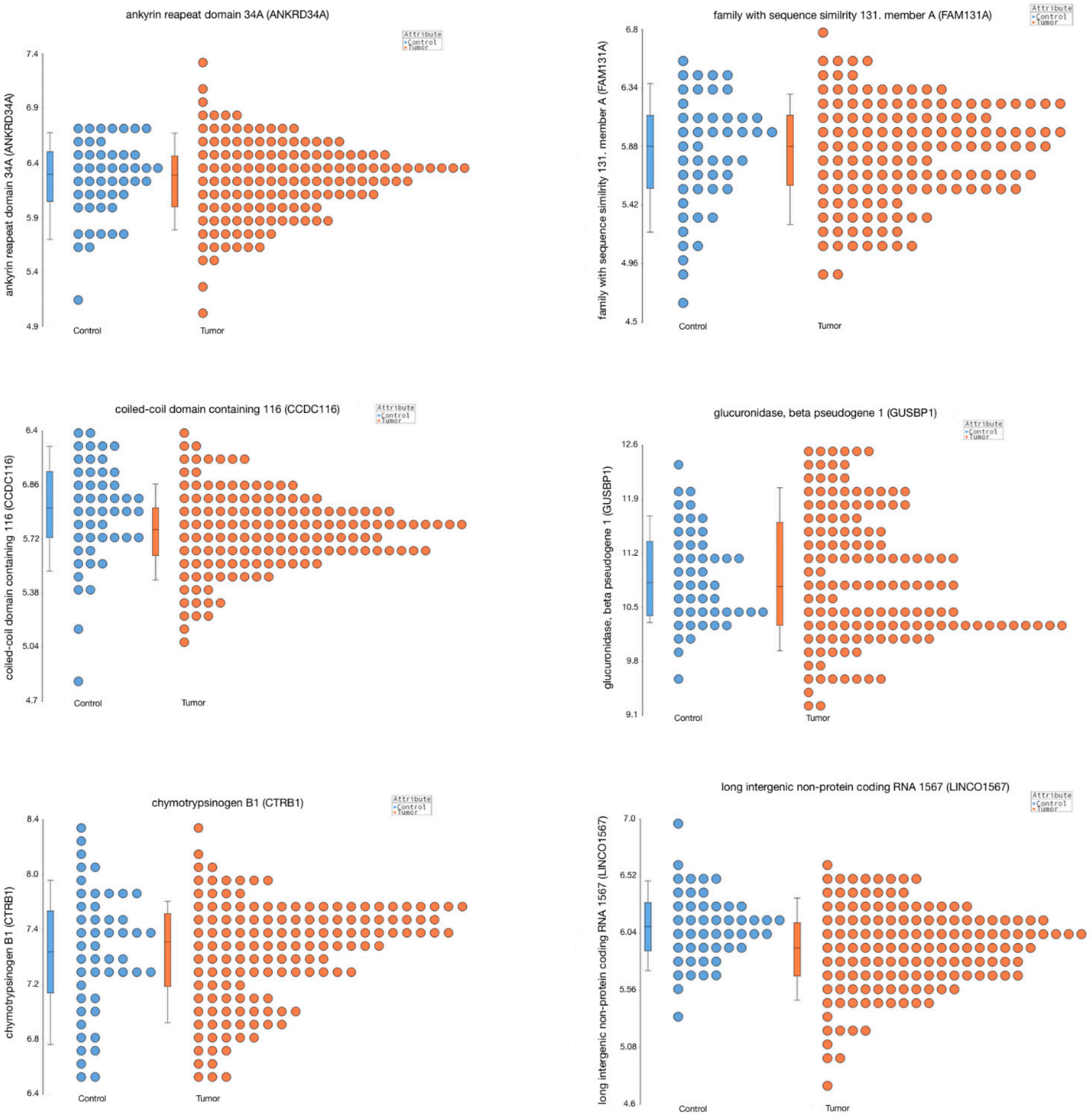

Figure S1. Six candidate genes that did not vary in gene expression in childhood cancer and healthy tissues. Dot plot of six genes with nonvarying expression level, *ANKRD34D*, *CCDC116*, *CTRB1*, *FAM131A*, *GUSBP1* and *LINC01567*. The blue dots represent healthy tissue and the red dots represent childhood cancer.

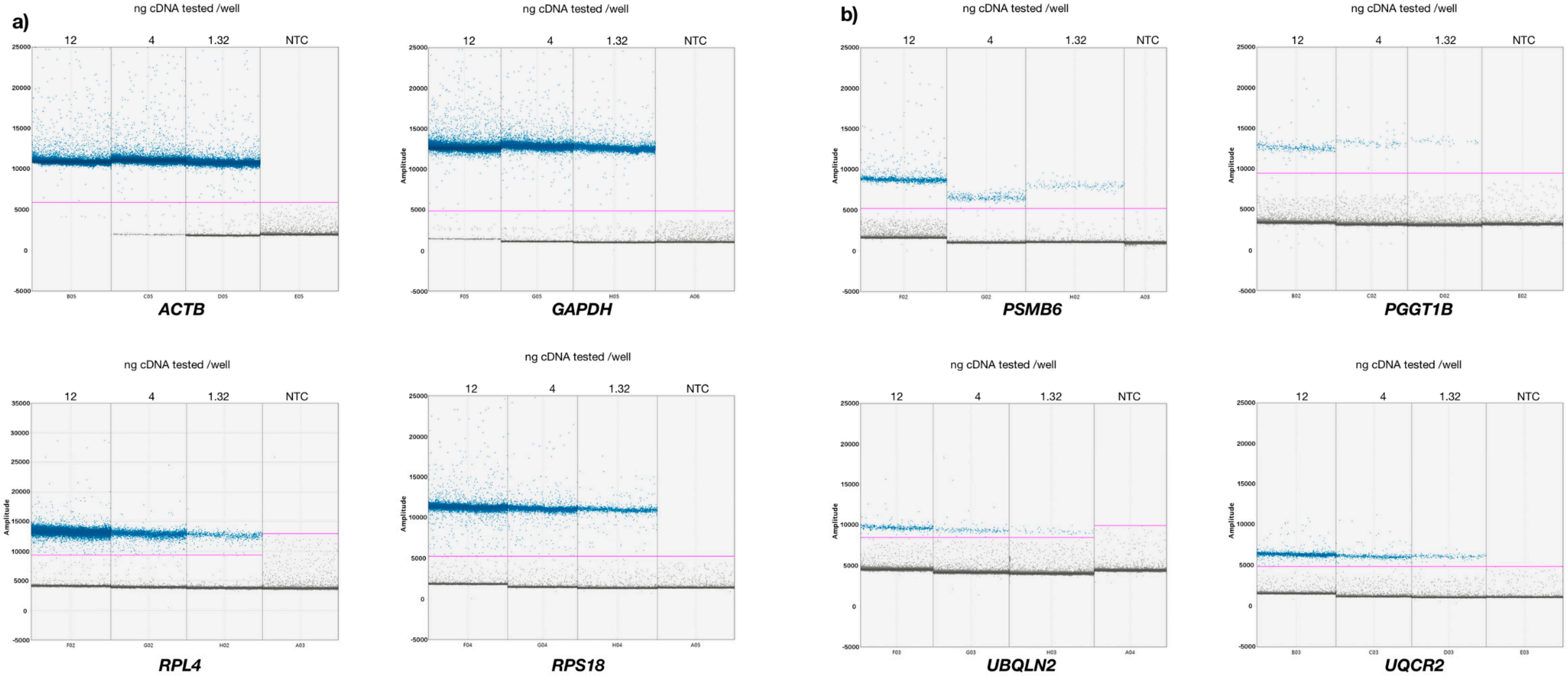

Figure 2. Amplification linearity of reference genes by ddPCR assay. **a**) ddPCR analysis plot showing linear amplification in serial dilutions of 1:3 of cDNA SUP-B15 for classical reference genes and **b**) new reference genes. Blue dots indicate positive amplification droplets and gray dots indicate negative amplification droplets. The pink line represents the cut-off of the positive and negative droplets.

Table 1. Database of childhood cancer microarrays for data mining of ArrayExpress.

Sample type ID	Number of microarrays
Osteosarcoma<br>GSE11414, GSE12865, GSE26244, GSE32395, GSE42903, GSE46549, GSE48281, GSE50532, GSE73166	125
Hodgkin lymphoma<br>GSE64232, GSE52710, GSE44244, GSE41493, GSE29545, GSE35224, GSE23591, GSE21296, GSE22550	172
Rhabdomyosarcoma<br>GSE15703, GSE22594, GSE27389	32
Retinoblastoma<br>GSE21147, GSE24673, GSE34379, GSE32381, GSE48989	41
Neuroblastoma<br>GSE26458, GSE34234, GSE34420, GSE59899, GSE78061, GSE66586, GSE56003, GSE34422, GSE51512	59
Medulloblastoma<br>GSE51020, GSE41842, GSE36947	31
Bone marrow<br>GSE47552, GSE34015	10
Skeletal muscle<br>GSE26276	3
Fibroblasts<br>GSE48761	10
Retina<br>GSE24673	2
Liver<br>GSE46960	31
Neuron<br>GSE65106, GSE56899	82
Leukemia<br>GSE48558	170

Table S2. Database of childhood cancer microarrays that passed quality control and were then used for the gene expression analysis.

Sample type	ID	Name of the microarrays
Osteosarcoma	GSE12865	GSM322691, GSM322692, GSM322693, GSM322694, GSM322695, GSM322696, GSM322697, GSM322698, GSM322699, GSM322700, GSM322701, GSM322702
	GSE48281	GSM1174178, GSM1174179, GSM1174180, GSM1174181
Hodgkin lymphoma	GSE29545	GSM731549, GSM731550, GSM731551, GSM731552, GSM731553, GSM731554, GSM731555, GSM731556
	GSE35224	GSM864079, GSM864080, GSM864081, GSM864082, GSM864083, GSM864084, GSM864085, GSM864086, GSM864087, GSM864088, GSM864089, GSM864090, GSM864091, GSM864092, GSM864093, GSM864094
	GSE23591	GSM578573, GSM578574, GSM578575, GSM578576, GSM578577, GSM578578, GSM578579, GSM578580, GSM578581, GSM578582, GSM578583, GSM578584, GSM578585, GSM578586, GSM578587, GSM578588, GSM578589, GSM578590, GSM578591, GSM578592, GSM578593, GSM578594, GSM578595, GSM578596, GSM578597, GSM578598, GSM578599, GSM578600, GSM578601, GSM578602, GSM578603, GSM578604, GSM578605, GSM578606, GSM578607, GSM578608, GSM578609, GSM578610, GSM578611, GSM578612
	GSE21296	GSM532266, GSM532267, GSM532268, GSM532269 GSM532270, GSM532271, GSM532272, GSM532273
Retinoblastoma	GSE21147	GSM529473, GSM529474, GSM529475, GSM529476, GSM529477, GSM529478
	GSE24673	GSM607938, GSM607939
Neuroblastoma	GSE26458	GSM649476, GSM649477, GSM649478, GSM649479, GSM649480, GSM649481, GSM649482, GSM649483
	GSE56003	GSM1350100, GSM1350101, GSM1350102, GSM1350103, GSM1350104, GSM1350105
	GSE34422	GSM848793, GSM848794, GSM848795
Medulloblastoma	GSE41842	GSM1025529, GSM1025530, GSM1025531, GSM1025532, GSM1025533, GSM1025534, GSM1025537, GSM1025544, GSM1025545, GSM1025546
Bone marrow	GSE47552	GSM1152350, GSM1152351, GSM1152352, GSM1152353, GSM1152354
Skeletal muscle	GSE26276	GSM645298, GSM645299, GSM645300
Fibroblasts	GSE48761	GSM1184255, GSM1184257, GSM1184261
Retina	GSE24673	GSM607947, GSM607948
Liver	GSE46960	GSM1142116, GSM1142117, GSM1142118, GSM1142119, GSM1142120, GSM1142121, GSM1142122, GSM1142123, GSM1142124, GSM1142125, GSM1142126, GSM1142127, GSM1142128, GSM1142129, GSM1142130, GSM1142131, GSM1142132, GSM1142133, GSM1142134, GSM1142135, GSM1142136, GSM1142138, GSM1142139, GSM1142140, GSM1142141, GSM1142142, GSM1142143, GSM1142144, GSM1142145
Neuron	GSE65106	GSM1587377, GSM1587406
Leukemia	GSE48558	GSM1180810, GSM1180813, GSM118016, GSM1180787, GSM1180791, GSM1180795, GSM1180798, GSM1180801, GSM1180804, GSM1180807, GSM1180818, GSM1180829, GSM1180841, GSM1180845, GSM1180769, GSM1180771, GSM1180764, GSM1180775, GSM1180808, GSM1180842, GSM1180785, GSM1180779, GSM1180780, GSM1180782, GSM1180811, GSM1180840
